# Court Decision on Vaccination

**Published:** 1917-02

**Authors:** 


### Court Decision on Vaccination.

The Third Court of Civil Appeals of Texas has recently rendered a far-reaching decision upon the question of compulsory vaccination. The substance of the decision is that city councils and school boards in cities and towns in Texas incorporated under the general laws of the State have no authority to enforce vaccination except in cases where an emergency exists for vaccination to prevent the spread of an epidemic.

The basis of the decision is that cities and towns have no powers except such as are specifically granted them by law, and that unless the law creating them gives them a right to enforce vaccination they cannot assume that right. Under the decision, it seems that even cities operating under special charters cannot resort to compulsory vaccination unless the charters granted by the Legislature specially confer that authority.

In a case decided by the Court of Civil Appeals at San Antonio some time ago, it was held that where there is an epidemic of smallpox, and vaccination is deemed necessary to prevent its spread, compulsory vaccination may be resorted to as an emergency measure. The recent decision does not disturb that opinion. Under this decision more school boards are powerless to compel students attending school to be vaccinated as an entrance prerequisite.

As the Legislature is in session, it is presumed that a bill will be introduced and pushed that will grant city councils and school boards the authority which they have heretofore been exercising, according to this decision, without authority. It is probable that the measure will be so drawn as to make certain the powers of all health authorities in such cases.

